# The Absence of Yellow Fever in Asia: History, Hypotheses, Vector Dispersal, Possibility of YF in Asia, and Other Enigmas

**DOI:** 10.3390/v12121349

**Published:** 2020-11-25

**Authors:** Goro Kuno

**Affiliations:** Centers for Disease Control and Prevention, Formerly Division of Vector-Borne Infectious Diseases, Fort Collins, CO 80521, USA; gykuno@gmail.com

**Keywords:** yellow fever, *Aedes aegypti*, Asia, vector dispersal, spillback, hypothesis, international sanitary convention, medical enigma, transmission cycle

## Abstract

Since the recent epidemics of yellow fever in Angola and Brazil as well as the importation of cases to China in 2016, there has been an increased interest in the century-old enigma, absence of yellow fever in Asia. Although this topic has been repeatedly reviewed before, the history of human intervention has never been considered a critical factor. A two-stage literature search online for this review, however, yielded a rich history indispensable for the debate over this medical enigma. As we combat the pandemic of COVID-19 coronavirus worldwide today, we can learn invaluable lessons from the historical events in Asia. In this review, I explore the history first and then critically examine in depth major hypotheses proposed in light of accumulated data, global dispersal of the principal vector, patterns of YF transmission, persistence of urban transmission, and the possibility of YF in Asia. Through this process of re-examination of the current knowledge, the subjects for research that should be conducted are identified. This review also reveals the importance of holistic approach incorporating ecological and human factors for many unresolved subjects, such as the enigma of YF absence in Asia, vector competence, vector dispersal, spillback, viral persistence and transmission mechanisms.

## 1. Introduction 

The accelerated occurrence of yellow fever (YF) epidemics in recent decades and the increased number of imported YF cases to permissive non-endemic countries, including China in 2016, have revived the century-old enigma of why YF does not occur in tropical Asia. Indeed, despite ideal ecological, epidemiological and demographic conditions, yellow fever virus (YFV) transmission has never been documented in the Asia-Pacific region [1,2,3,4,5,6,7,8,9,10]. Surprisingly, the rich history of human intervention to prevent YF in Asia that lay behind this enigma and which is a major factor indispensable in the debate has never been brought to attention. Furthermore, proposed hypotheses have not been comprehensively analyzed in light of accumulated recent research data. 

Africa is considered the birthplace of YF. Because YFV dispersed out of Africa to the New World in association with the slave trade between the 16th and 19th centuries [11] and some members of the YFV group of viruses, such as Wesselsbron, Edge Hill, Sepik, Fitzroy River and Bamaga viruses, occur in Asia, New Guinea, and Australia [12,13], the absence of YFV in tropical Asia and the Oceania is indeed puzzling. Thus, identifying the factors responsible for the absence of YF in Asia could provide valuable information to prevent and control YF in other regions of the world infested by the vector but still without YF. Here I focus first on the historical events and preventive measures undertaken in the past, then examine several major hypotheses proposed to explain the enigma, unresolved issues on such subjects as vector dispersal, persistence in urban environments, and spillback mechanism, by emphasizing the problems and unknowns to identify research questions. 

## 2. Information Retrieval 

While most journal articles are readily retrievable from multiple electronic databases available online, hidden historical information is far more often discovered in books and book chapters rather than in journal articles, thus requiring a two-stage search. In the first stage, books and book chapters were identified by applying a broader range of related or intertwined subjects as probing keywords. This was performed by Google search. In the second stage, the books or chapters identified were read, and more specific combinations of keywords were selected to further specify the range of search, and relevant documents identified again using Google search. Finally, the desired original sources were secured and read. 

## 3. Historical Events

In the history of YF that scourged not only parts of the tropics but multiple temperate regions of the world, the discovery of Juan Carlos Finlay of Havana, Cuba that *Aedes aegypti* is responsible for the transmission of the etiologic agent was pivotal for further advancement of the research by a team of the U.S. Yellow Fever Board headed by Walter Reed at the turn of the twentieth century [14]. The Finlay’s contribution, Reed Commission’s discoveries of the mechanism of transmission and filterability of the agent, and William Crawford Gorgas’ successful elimination of YF by a rigorous campaign to destroy the mosquito have been immortalized in the history of medicine. Though these early achievements were collectively hailed as the major triumph, certainly they did not signal a sign of conquest, as abundantly revealed in a comprehensive review of subsequent research by dedicated workers around the world [15]. In fact, more than a century later, this formidable enemy is still shielded by multiple layers of enigmas and refuses to surrender.

Shortly after the success in YF control in Cuba, the U.S. decided to undertake construction of the Panama Canal which had been abandoned earlier by the French because of a huge loss of life due to multiple diseases including YF.

**Figure 1:**
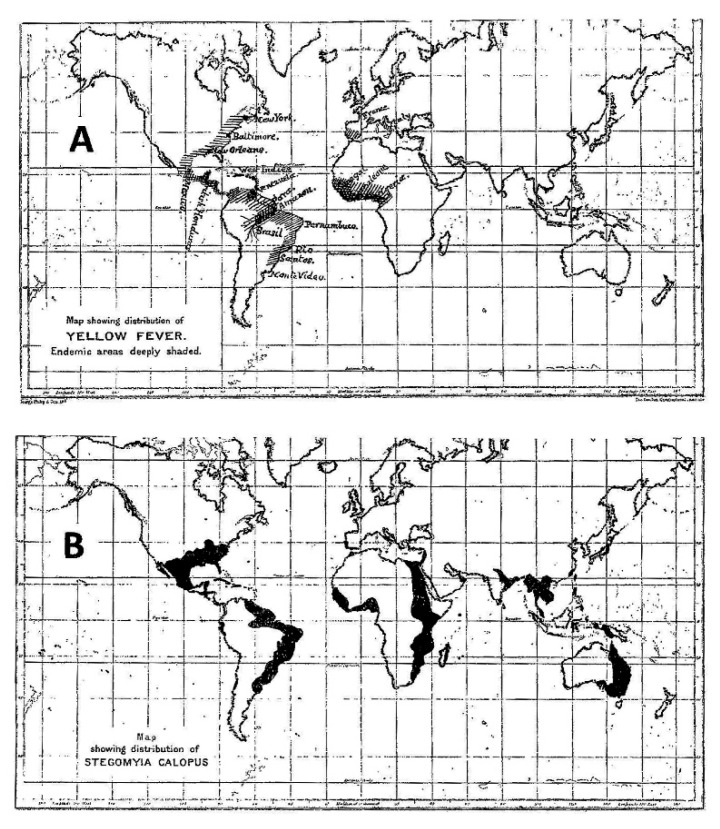
Geographic distributions of yellow fever (**A**) and of *Ae. aegypti* (**B**) (Source: Boyce, 1911; with permission).

At the beginning of the 20th century, with the widespread geographic distribution of *Ae. aegypti* in Asia in the background, completion of the Panama Canal significantly increased the risk of spreading YF. But, a puzzling discrepancy between the geographic distributions of this vector and of YF was cartographically recognized in 1911 by Rubert Boyce (Figure 1), showing absence of YF in Asia, despite occurrence of the vector [16]. Boyce, on the other hand, while acknowledging the fact that YF no longer occurred in North America and Europe at the time of his writing, nevertheless included these regions on the map because of historical records. 

### 3.1. Patrick Manson’s Speech

On the eve of the commencement of the second attempt to construct the canal on 25 February, 1903, Patrick Manson addressed a meeting of the Epidemiological Society in London, where he made an impassioned speech about the imminent threat of the invasion of YF to Asia once the Panama Canal was completed [17]. His points of emphasis during the speech are summarized as follows:

1. The direct trade and human travel between Asia and YF-infested parts of the Americas, which will be made possible upon completion of the Panama Canal, will introduce YF to Asia where the principal vector mosquito is abundant.
2. Once YF is introduced to Asia, it will spread among major urban areas, such as Hong Kong, Shanghai, Bangkok, Singapore, Batavia (now Jakarta), Colombo, Bombay (now Mumbai), and other cities, since people there totally lack immunity.
3. The Canal opening will result in decimation of the population similar to that caused by European diseases introduced to the Americas after Christopher Columbus discovered the New World.
4. A sanitary organization must be established prior to completion of the Panama Canal to prevent dispersal of YF. Sanitary officers must be empowered to board ships to inspect conditions, quarantine anyone suspected of having YF, fumigate to eliminate mosquitoes, and to destroy mosquito eggs.
5. An international agreement on health matters must be established between governments to prevent dispersal of YF from endemic regions of the world.
6. During outbreaks of YF, there may be undesirable activities, such as dissemination of distorted information, dangerous experiments using the etiologic agent, and trafficking of infected mosquitoes.

### 3.2. YF Elimination in the Panama Canal Zone

The impact of Manson’s speech and a variety of proposals raised among the participants during the discussion that immediately followed after the speech were clearly illustrated by the fact that William C. Gorgas (known for his success in eliminating YF in Havana, Cuba with a vigorous mosquito control campaign) was appointed as head of all health matters during the construction of the Canal and that Henry R. Carter (known for discovery of the “extrinsic incubation period” in mosquitoes) was assigned to a post for the inspection of the ships and quarantine for YF control [18]. 

### 3.3. YF in Hawaii

Manson’s concern was realized even before the completion of the Canal [19]. Because of the experiences with non-indigenous disease epidemics brought by foreigners after the Captain James Cook’s visit to Hawaii, the U.S. Public Health and Marine Hospital Service conducted strict inspection, disease diagnosis, and quarantine of vessels arriving in Hawaiian waters. The first case of shipboard YF was detected in Honolulu in 1910, but quarantine prevented further spread. A year later, the same cargo ship carrying a sick passenger (who boarded at Callao, Peru) with a stopover in Mexico arrived in Honolulu. The illness of the shipboard patient was diagnosed as YF, and quarantine guards were immediately posted on board while the ship was thoroughly fumigated. However, one of the guards posted on board during fumigation became sick with YF seven days after the arrival of the ship. The patient and all residents who lived near the index case were quarantined, all houses were fumigated three times at intervals of 24 h for 8 days, and the circular area surrounding the residence of index case (100 yards in radius) was completely stripped of vegetation and treated with insecticide. Furthermore, a barbed wire fence was erected around the area and the area guarded by the U.S. Army staff to restrict human movement for 90 days [20]. Because of the prompt and meticulous actions, no secondary cases were detected. Thereafter, all vessels arriving in Honolulu from any YF vector-infested locations were fumigated before being permitted to enter the port [19,20].

### 3.4. Yellow Fever Investigation by the Rockefeller Foundation

In 1914, shortly before the completion of the Panama Canal, Wickliffe Rose, the newly appointed Director of the International Health Division (IHD) of the Rockefeller Foundation (RF), visited Singapore, Malaysia, India and the Philippines in search of medical research projects worthy of investing a huge sum of money for humanity. While in Singapore, he was told of a serious concern of the British authorities in India regarding increased risk of introducing YF to Asia because of the Panama Canal [21,22]. Henry R. Carter, when consulted by Rose, felt Manson’s concern was an exaggeration but still felt that taking preventive actions in Asia was appropriate. Rose then requested William C. Gorgas to lead a YF investigation for the RF [21]. Gorgas held a firm belief at the time that YF was an urban problem which could be eradicated [23]. “Jungle yellow fever” was unknown at the time. In the following 35 years, the RF researchers isolated YFV and many other viruses associated with arthropods and wildlife. Accordingly, although the discovery by the U.S. Army Board in Cuba was the principal reason for the chain of events that resulted in the birth of arbovirology as a unique branch of viral disease research, the warning of Patrick Manson in 1903 provided a critical stimulus for the chain of events to evolve faster [17,24,25,26,27]. 

## 4. Measures to Prevent YF Dispersal

### 4.1. International Conventions

The discovery of YF transmission by mosquitoes in 1900–1901 stimulated establishment of the first international organization dedicated to international control of disease dispersal, the International Sanitary Bureau, by the eleven American states in 1902 [28]. This and Manson’s speech in 1903 led to the International Sanitary Conference held in Paris in 1903, where YF was provisionally added to the list of quarantinable diseases (cholera, plague, smallpox and typhus) that required a coordinated international intervention to control spread. The use of quarantine as a means of preventing YF spread after 1903 was significantly different from the earlier use of lazarettos in Europe and the Americas in terms of rationale, method used to segregate people arriving from YF-infested locations, practice of inspection or fumigation of the vehicles used for transportation (ships in early days but also aircraft later) and inspection of the cargoes on board. In the former, lazarettos were used indiscriminately for the persons arriving from any YF-infested regions when the mode of transmission was totally unknown, while in the latter the measures adopted were more specific because mosquito-borne transmission was known. 

Table 1 shows selected early events where international prevention of YF dispersal was a major topic before the establishment of the World Health Organization (WHO). The table emphasizes the early prevention of YF dispersal to Asia. Initially, maritime navigation, sharing disease intelligence, quarantine, mooring requirements, ship inspection and fumigation were emphasized. By the late 1920s, aircraft became a potential means of disease dispersal, and the importance of quarantine at sea ports began to lose support. The concept of YF prevention strategies for international transportation by aircraft was proposed by the Office International d’Hygiene Publique that had been established in Paris. The concept required establishment of specially designated airfields called “antiamaryl aerodrome,”each with a wide swath of its perimeter to be maintained vector-free at all times (Table 1). All “anti-amaryl aerodromes” had facilities for medical examination of crew and passengers, quarantine, dwellings for crew and maintenance staff, and capacity for aircraft fumigation [29]. Disinsection (elimination of live mosquitoes in the aircraft cabin with insecticide) was ratified in the International Sanitary Convention of 1933 and modified in the 1944 Convention [30]. This practice was also applied to flying boat services in Africa from the 1930s through early 1940s, in particular in Kisumu and Naivasha, Kenya which served as important hubs for travel to the Middle East and to Asia. The objective was to prevent dispersal at the point of embarkation in YF-endemic areas before arrival at a designated anti-amaryl aerodrome in Asia. After the WHO was established, the requirement for YF vaccination certificate for international travelers replaced the maritime and aerodrome regulations. 

Elsewhere, including the Americas, Europe, and much of Asia, most countries accepted either the 1933 international convention for aerial navigation and/or its 1944 modified version. However, in these regions no anti-amaryl aerodrome was established and the airports designated “sanitary” meant nothing more than provision of medical service for travelers without a specific reference to YF inspection. Still, before full implementation of vaccination requirement by the WHO, at ports of entry near the southern border of the United States, medical inspection of passengers and fumigation of the aircraft (including flying boats) arriving from YF-infested or *Ae. aegypti*-infested tropical countries were rigorous; and even monkeys imported from tropical countries were inspected at seaports.

As shown in Table 1, the measures adopted to prevent YF spread at most international conferences were primarily focused on the regions between Africa and India and changed multiple times over the period, largely because of politics, shift in the level of medical concern, and changing means of international travel or transportation. Despite changes in regulations over time, total absence of YF symptom while in a YF-free area for a minimum of six consecutive days immediately before departure or upon arrival, which was required for international travelers, was universally accepted in all sanitary conferences since its first official adoption at the 1909 convention of the International Sanitary Bureau (later Pan American Health Organization or PAHO) held in Costa Rica for the countries of the Americas. 

After the Patrick Manson’s speech in 1903, British authorities continued to devise measures to prevent dispersal of YF, and Manson’s views were widely disseminated to all levels of government, including the Colonial Office. Although not all British diplomatic staff were in favor of strict measures to prevent YF dispersal, measures such as total embargo of flights from YF-endemic West Africa to “seemingly YF-free” East Africa, were discussed by the British Medical Association in 1932 [31]. The railway links from West Africa to Central and South Africa were also identified as another sources of concern for the spread of YF. 

### 4.2. India and East Africa

In India where YF did not occur and when its transmission mechanism was totally unknown then (1796), oddly a British physician (Charles MacLean) working for the British East India Company in Calcutta (now Kolkata) advocated against the use of lazaretto for YF control as useless and received a wide attention in and outside India [32]. The preventive activities undertaken in India and her coordinated activities with African countries are inseparable, as described below. 

Among the British colonies in Asia, the aforementioned Manson’s message was most seriously received in India. By 1910, the Indian Branch of the British Medical Association called for an international campaign against *Ae. aegypti* in YF-endemic countries. The Government of India added YF to the list of quarantinable diseases and implemented port regulations including port sanitation and mooring requirements. In 1912, a military surgeon, Sydney P. James was dispatched to the Americas on a fact finding mission to assess the possibility of YF dispersal to Asia. James visited the Panama Canal, Havana, Guayaquil, New Orleans, and Honolulu, acquiring a massive amount of information about the pattern of YF spread and the measures adopted to control YF [20,33]. 

Although James, like Carter, did not entirely agree with Manson’s warning and discounted the possibility of YF spread to India because of the long distances from the Americas, he thought that eastern parts of Asia, such as Hong Kong and Singapore, were vulnerable and also recommended India to reduce vector populations. Furthermore, he proposed that British India Medical Service officers be stationed at the Panama Canal, Hong Kong and Singapore for monitoring the occurrence of YF to enable rapid communication with all concerned authorities in Asia [20,34]. His justification of negligible threat to India due to logistics, however, was disputed by others because YFV could survive for long periods aboard ships by cycling between mosquitoes and humans. There was also a strong opposition and reluctance by the provincial governments in India and Great Britain to implement the maritime regulations because of high cost, lack of resources, inconvenience and trade/travel disruptions. Although the administrative power of India was limited because it was still a part of the British Empire [33], India still stressed at international conferences (i) protection of ports, (ii) elimination of vectors at ports, and (iii) imposition of strict quarantine practice for the prevention of YF importation. This reflected the fact that by the mid-1920s the source of YF had shifted from the Americas to Africa, and the principal means of international navigation was shifting from ships to aircraft. Khartoum of Anglo-Egyptian Sudan served as a major hub for air traffic from YF-endemic West Africa to the Middle East and British India. At the 1932 Conference in Cape Town, South Africa, India negotiated with Anglo-Egyptian Sudan and governments of West Africa for Sudan to serve as a “buffer zone” to prevent introduction of YF [33]. At the conference, Wilbur A. Sawyer of the RF suggested that the mountainous lake region of East Africa was apparently serving as a natural barrier against the spread of YF to the East [34], only to find out shortly that the region had been already YF endemic [35]. 

A set of requirements in this agreement included medical examination of all passengers and crew for YF and fumigation of aircraft at a designated “anti-amaryl (YF) aerodrome” [29]. The requirement for passengers to board was total absence of YF symptoms for 6 consecutive days in the YF-free area immediately prior to boarding. Only certified aircraft and passengers were allowed to depart for another “anti-YF aerodrome” located in Asia. “Anti-YF aerodromes” were built after 1933 in Kano (Nigeria), Malakal (South Sudan), Accra (Ghana), Anglo-Egyptian Sudan (four airfields) and Karachi (now of Pakistan). “Sanitary aerodromes” which did not meet the requirements of anti-YF aerodrome, but which were located in Belgian Congo (now Democratic Republic of Congo), Cameroon, Congo, Chad, Djibouti, Egypt, Ethiopia, Gabon, and Ghana, at least for short periods screened suspected YF passengers. As an additional safeguard, India, Iraq, and Egypt agreed to send alerts by telegraph of incoming flights from YF-infested areas. 

When YF spread to Eastern Africa was serologically confirmed by the RF researchers in the early 1930s, the fear of YF close to home was so strong in Kenya that the government did not allow establishment of a YF laboratory for further studies as proposed by the RF. Thus, a continuing study was conducted at the YF Laboratory in Entebbe, Uganda established in 1936. Belgian Congo also acted similarly by refusing establishment of a YF laboratory by the RF. The major outbreak in the Nuba Mountains in southern Sudan in 1940 made it necessary to prevent dispersal of vectors to Europe from East Africa, by requiring changes of aircraft at Alexandria and Khartoum for all types of aerial services. This requirement was replaced by a new convention ratified in 1948 among 14 countries of the United Nations. However, the anti-YF aerodromes in Sudan functioned until 1951. As mentioned earlier, unbeknownst to the delegates of the Cape Town conference at the time, by the time this buffer zone system was established, a serosurvey had shown that the regions in and around Sudan had been already YF-endemic, characterized by “silent” transmission without overt epidemics [35]. After YF vaccine became available in the late 1930s, strict regulations, including proof of vaccination, were put into effect to prevent introduction of YFV by air and by land in Anglo-Egyptian Sudan, Belgian Congo, Kenya, and Uganda.

The Government of India outlawed possession of YFV in 1931 [33]; but in the early 1940s, the U.S. Government approached the Government of India with a proposal for establishing a YF vaccine manufacturing capacity, the rationale being that such a facility was necessary because the Japanese Army might apply biological warfare using YFV in the event of war in tropical Asia. Coincidentally, in 1940 a large YF epidemic affecting nearly 40,000 people (with more than 1500 deaths) occurred in Anglo-Egyptian Sudan [36]. The Government of India accepted the offer and sent Chitaman G. Pandit to the RF laboratory in New York to learn the manufacturing technique from Max Theiler and George K. Hirst. Eventually, a facility was built in Kasauli [37]. However, India had to suspend manufacturing in 2008 because of mechanical breakdown. As of 2020, India needs to import YF vaccine. Nevertheless, before China began manufacturing the vaccine for domestic consumption recently, India had been the only country in Asia with a YF vaccine manufacturing facility in the region [38]. 

India continued to propose stringent requirements for preventing YF dispersal at WHO meetings, demanding a more precise definition of “YF-endemic” and “YF-receptive” areas, first in the International Sanitary Regulations and later in the International Health Regulation (IHR). IHR obligated all countries to report quickly any case of YF. In 2011, with an encouragement and organizational help from John P. Woodall and Duane J. Gubler, India and the WHO sponsored a meeting in Goa to encourage Southeast Asian countries to develop contingency plans for the introduction of YFV to the region. Although most countries in this region developed an emergency response plan at the time, the current status is uncertain. Unfortunately, in contrast to the early days of history, countries in the region (with the exception of Pakistan) generally did not share India’s fear of YF in later periods [39]. As of 2020, among Asian countries, India still requires the strictest YF vaccination proof for selected arriving international travelers.

### 4.3. Other Asian Countries

Although YF never occurred in Asia, the disease was called colloquially in Martinique “mal de Siam” when an outbreak occurred there in 1648 after the arrival of a French naval ship originating in Thailand, because people in Martinique did not know the significance of the fact that the ship made a call at the YF-endemic Recife of Brazil prior to the arrival at Martinique. A theory of Asian origin of YF by George Augustine was based on this equivocal belief solely because that outbreak occurred a few months before another outbreak in Yucatán, Mexico, which had been known to be the earliest incident in the Americas at the time [40]. In Hong Kong, a mysterious “bilious fever” outbreak characterized by yellowing of the skin and “black vomit” in some patients occurred in 1865–1866 [41]. Naturally, it aroused a suspicion of YF, but the etiology was not identified. However, because of this episode, quarantine regulations in Hong Kong were later amended to cover YF [34]. 

In Asia, international meetings on tropical medicine were held, and other scientific activities were conducted. In 1912, a conference concerning YF was held in Hong Kong during which a set of preventive measures was proposed by James. The set included addition of YF to the list of quarantinable diseases in Hong Kong and Singapore. In the following year, Johannes van Loghem, representing the Dutch East Indies, proposed at the 17th International Conference of Medicine in London creation of disinfecting stations at ports in East Asia under international control. To this proposal, Manson suggested the Netherlands to initiate a diplomatic negotiation first with the USA and later with colonial powers in Asia. As a result of a chain of negotiations, Britain, the Netherlands, Germany, France, and Portugal began a communication regarding establishment of anti-YF measures in the region [34]. Prohibition of importing YFV for any reason including research among all countries was instituted through gentleman’s agreement in early 1930s.

The Far Eastern Association of Tropical Medicine was created in 1908 in Manila at the suggestion of Victor Heiser of the RF [42] and held biennial meetings for countries in East Asia, Western Pacific and Australia until 1938. At the meeting of this association at Batavia (now Jakarta) in 1921, the government of the Dutch East Indies reiterated her concern over the YF threat through the Panama Canal and initiated an experiment to reduce vector population by installing water mains in a major port city. In another meeting at Tokyo in 1925, Wilhelm H. Hoffman, a German physician and director of the Finlay Institute, Cuba, confirmed that Asian and American *Ae. aegypti* were identical [43]. At another meeting in Bangkok in 1931, prohibition of importation of infectious YFV to Asia for any reason including research and a proposal to establish “anti-amaryl aerodromes” as sanitary enclaves in all capitals in the region were discussed. Emilius P. Snijders of the Netherlands presented the results of YFV susceptibility tests using Asian monkeys, vector competence of Asian mosquitoes and lack of evidence of vertical transmission of YFV in *Ae. aegypti*. He concluded that cross-protection among dengue and YF infections was “relative,” despite some supporting observations elsewhere [44,45]. The League of Nations set up the Eastern Bureau (within the International Health Office) in Singapore with a financial support from the RF and began wireless communication of current disease information for the first time in the world for an international medical surveillance. 

Dutch East Indies: In the colonial days of the Dutch East Indies, ship-board physicians were required to report all types of diseases observed during the journey; the passengers deemed dangerous (including suspected cases of YF) were quarantined; and the penalty for failing the regulation was heavy. However, because the ships serving the Dutch East Indies Company sailed south of the Indian Ocean far away from East Africa, and stopped for supplies only at YF-free offshore African islands and Cape Town, there was little possibility of introducing YFV to the Dutch East Indies. In 1908, Johannes van Loghem made call to arm against YF spread and continued to engage in preventive activities in the next 2 decades. In the late 1930s, the Dutch East Indies prohibited aircraft landing for the flights originating in Africa.

### 4.4. US Army During World War II

During World War II (WWII), in the Burma Campaign by the British India, nearly 100,000 East and West African colonial troops participated, but YF did not occur, possibly because of the activities of the U.S. Army to prevent spread of YF during WWII. In the Africa-Asia areas, the East Africa Command engaged in complex, multiple international arrangements to prevent YF spread through activities of the Preventive Medicine Service of the U.S. Army, then headed by James S. Simmons. The organizations participating in these collaborative activities included the RF, other scientific organizations, civilian and governmental agencies (such as U.S. Public Health Service, Department of State, the British Inter-Departmental Committee on Yellow Fever Control, Governments of India, Egypt and Brazil) as well as other U.S. Military commands in North Africa, Middle East, Caribbean, China-Burma-India, and Pacific Ocean areas [46]. Nearly 7 million doses of YF (17D) vaccine produced by the RF were administered to US and British troops. Because of the inclusion of human serum (from a blood donor infected with Hepatitis B virus in one lot) used as a stabilizer, nearly 27,000 cases of hepatitis occurred [47]. This problem was quickly solved by switching to a vaccine production without use of serum as stabilizer at the Rocky Mountain Laboratory in Montana. Also, it turned out that Japanese Army did not possess infectious YFV during the war. As a result, no case of YF occurred among military and civilians involved in war-related activities. Thus, the validity of one case of YF registered in Hong Kong in 1945 [48] is highly questionable. 

### 4.5. French, British and RF Activities

More than 15 million doses of a vaccine produced by the Institut Pasteur using a French strain were administered through a compulsory program to civilians and military personnel by scarification primarily in French West Africa (Senegal and Ivory Coast) but also in Sudan between 1939 and 1945 [49]. In the U.K., Edward Hindle at the Wellcome Bureau of Scientific Research in London engaged in a variety of YF research including development of a killed YF vaccine since late 1920s. 

“The absence of YF in Asia” also played an important role in the YF research conducted in Europe and in West Africa by the RF. The poorly recognized but extremely valuable history behind the major event in the history of YF is briefly presented. During a visit to Trinidad in 1914, Andrew Balfour of the U.K. learned of an intriguing information that high mortality of howler monkeys often coincided with an outbreak of YF there [50]. Then, Andrew Connal of the British YF Commission proposed to use howler monkeys for research. However, the British attempt was unsuccessful, because those neotropical monkeys could not adapt to life in captivity. Later, Oskar Klotz (a Canadian physician and microbiologist with an experience in YF investigation in Brazil and who was hired by the RF as an adviser for the RF’s YF investigation in Africa) theorized that Asian monkeys would be better substitutes for howler monkeys because Asian NHPs had not yet developed immunity to the agent of YF in contrast to African NHPs which had been exposed to such a host-parasite relationship for centuries. Besides, ready adaptation of many Asian monkeys to confined conditions was well known among zoo keepers. Furthermore, Klotz knew that rhesus monkey had been used for YF research earlier [51,52,53,54,55,56]. 

Klotz suggested Frederick F. Russell (then director of the IHD of the RF) to abandon guinea pigs and African monkeys and use Asian monkeys instead, to break the RF’s long-stalled YF investigation. Though reluctant at first, Russell finally agreed, and Henry Beeuwkes (the director of the YF laboratory in Nigeria) arranged to import *Macaca mulatta* (commonly known by rhesus monkey) and *M. sinica* from animal dealers (Chapman in London and Hagenbeck in Hamburg, Germany) [51,52]. The subsequent events including the tragic death of Adrian Stokes (a British physician on leave from Guy’s Hospital in London who participated in YF research as a member of the RF) and successful isolation of YFV (a strain from a Ghanan patient named Asibi) in 1927 for the first time have been immortalized in medical history. After this episode, Asian monkeys were used in all YF laboratories in Europe, Africa, North America and South America.

## 5. Proposed Hypotheses to Explain Absence of Yellow Fever in Asia 

Once there existed in Asia a myth that dengue was a mild form of YF. It was put to rest in early 1930s. Thereafter, many overlapping hypotheses have been proposed to explain the enigma of YF absence. 

### 5.1. Distance to Asia and Scarcity of Opportunity

With only one example of an epidemic of unknown origin reported in 1865–1886 in Hong Kong (as described in Section 4.3), near total absence of written documents or legends referring to an episode of epidemic disease compatible with YF and the lack of any laboratory-confirmed case of YF in individuals presenting a clinically compatible YF syndrome anywhere in Asia support the conclusion that YF has never circulated in Asia [57]. The unstated assumption of most researchers of this question has been that this was due to lack of opportunity for YFV introduction [17]. Thus, the combination of long distance and travel time by ships from YF endemic countries in Africa and the Americas to Asia was prohibitive for the survival and introduction of YFV [17,58]. Also, the low volume of travel and trade between YF-infested part of Africa and Asia, compared with the enormous volume of travel and trade between West Africa and the New World, further decreased the probability of YFV introduction to Asia. 

Some have speculated that unrecognized sporadic minor YF outbreaks could have occurred in Asia but left no trace or record, since reliable serologic tests for confirmation had not been developed until early 1930s. Serosurveys by a neutralization test revealed that residents in Asia did not have antibodies to YFV, although a small number of positive reactions were observed in India. These puzzling results were interpreted to be the result of non-specific cross reaction with other endemic flaviviruses [59,60]. A serosurvey of nonhuman primates (NHPs) in Malaysia similarly revealed no evidence of YF infection [61]. 

### 5.2. Human Transport

**Figure 2:**
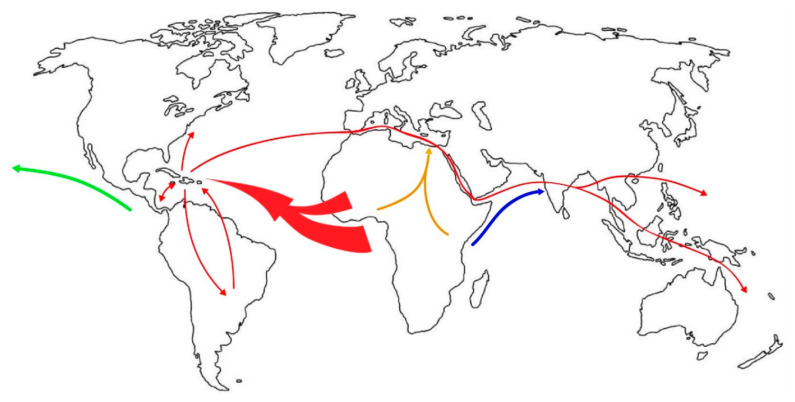
Possible routes of *Ae*. aegypti dispersal to tropical Asia from Africa. (Red: As proposed in [119] by Powell et al.; Blue: East Africa route; Green: From the Americas to the Pacific; Orange: Inland dispersal to the Middle East).

That slave trade across the Atlantic between West Africa and the New World as a means of YF dispersal is well recognized (Figure 2, thick red arrow). On the other hand, the volume of slave trade between West Africa and Asia was far less, which is thought to be a factor contributing to the absence of YF in Asia [62]. However, there was human transport (including slave trade as well as transport of indentured laborers) across the Indian Ocean between India and East Africa. Although the volume of human transport per year across the Indian Ocean was lower than that across the Atlantic, particularly during the height of slave trade to the New World [63], the cumulative volume over several centuries was actually greater between East Africa and Asia because the trades to and from Asia across the Indian Ocean lasted much longer. However, the paucity of YF in East Africa greatly decreased the probability of introducing YF to Asia. After the British abolition of slavery in 1833, there was an acute shortage of workers in the British colonies, and large numbers of Asians consisting mainly of Indians, Chinese, and Malays were recruited to work in diverse locations, including parts of Africa, Mauritius, Fiji, and YF-infested West Indies, Central and South America. During the construction of the Panama Railway and the Panama Canal, large numbers of Asian workers were reported to have died of YF, although there was no official or reliable documentation of the cases, with the exception of 3 cases of Japanese laborers admitted to the Ancon Hospital (previously L’Hopital Central du Panama by the French) in Panama during the American construction of the Canal. There was little or no return travel of those migrants back to their home countries because most impoverished migrants could not afford the fare, decreasing the risk of introducing YF to Asia.

### 5.3. Human Genetics

Racial differences in susceptibility to YFV infection likely gained support early in history because of a prevalent belief that YFV and humans co-evolved in Africa [64] and because of the observation that the disease was more severe and had a higher case fatality rate (CFR) in Caucasians than in black people. However, Boyce believed that the resistance of Africans was due to acquired immunity rather than inherited character [16]. That all races are susceptible to YFV infection is supported by the fact that severe disease and death have been reported in Japanese, indigenous “American Indians” in Panama and Brazil [65,66], “East Indians” (descendants of the immigrants from India) in Trinidad [67,68], Lebanese in West Africa (Senegal and Sierra Leone) [69], and Chinese in 2016. 

However, a recent study based on epidemiologic records of YF outbreaks in the 19th century in the U.S. [70] revealed a significant difference in CFR between Caucasians (CFR: 25.0–72.5%) and blacks (CFR: 1.1–14.1%). Since this topic has been a highly controversial biomedical issue for many years [71,72,73,74,75], it deserves a re-examination. The CFRs in Africa in modern period were near 20% [76,77,78]. Because there exists a hypothesis of cross-protection by flavivirus exposure prior to YF infection (Section 5.5), given a high prevalence of flavivirus infection there, there is a possibility that CFR in those without such a prior exposure could be even higher. At any rate, if YF outbreak occurs in multi-racial communities in the future, application of advanced genetic profiling techniques may help reducing involvement of bias and other problems. Regardless of the outcome, genetic factor does not explain YF absence in Asia.

### 5.4. Variation in Mosquito Vector Competence

Variation among geographic subpopulations of the YF mosquito vector, *Ae. aegypti*, in YFV transmission was observed early in history. Jean Legendre advanced a theory that there exist multiple “races” of the vector [79], which he used to explain absence of YF in Asia. This stimulated research on the variation of vector competence among geographic strains of the vector. Sheldon F. Dudley went even further, postulating complete lack of transmission capacity of the Asian strains of *Ae. aegypti* to explain YF absence [80]. Although lower competence of Asian strains has been reported, the difference was not found to be compelling [81]; and at least under laboratory conditions, some Asian strains were more efficient than those from the YF-endemic areas of Africa or South America [82,83]. 

A major problem in assessing the importance of vector competence in studying the dispersal of YFV to new territories or variation in transmission rate has been the criteria used to define the concept. Although there are many parameters, only selected parameters directly related to vectors have been selected in simplified laboratory experiments for convenience due primarily to the large number of variables involved and a complication associated for designing an experiment. Lack of standardization of experimental protocol, lack of universal reference virus (low passage), and questionable relevance of the host chosen are just some examples of the problem [84]. Use of YFV(17D) may be important for evaluating attenuation but the strain is irrelevant for vector competence testing. Use of long-colonized mosquito is another source of problem [64]. Some factors are not included because they are not readily quantifiable for comparison. As a result of this complexity, sometimes result of vector competence test may be perplexing. For example, *Ae. aegypti* obtained from a region where a YF epidemic occurred just a short time earlier was found to be incompetent in one report [85]. 

Actually, vector competence is more complex than that and includes viral persistence mechanism(s) such as vertical transmission in the mosquito, innate immune mechanisms of vector, competition between commensalistic viruses or endogenous viral elements and arboviruses, virus-encoded mechanisms for facilitating replication in vectors (such as subgenomic noncoding RNAs) [84,86]. Furthermore, in natural conditions, vector competence is more broadly defined as the sum total of complicated interactions among factors associated with virus, vector, and host in a given environment. All of these mechanisms potentially impact not only on vector competence but on viral dispersal, persistence, and transmission dynamics. But, these topics are not discussed because they are beyond the scope of this review.

### 5.5. Cross Protective Immunity in Humans

In 1923, Cornelis Bonne in Suriname conceived a hypothesis that prior experience with a dengue-like illness would modulate the severity of YF [34]. This was received favorably in the Dutch East Indies where dengue was prevalent [45]. Actually, this phenomenon had been observed earlier in the 19th century when indentured Indians and British troops who had previously served in India were found less susceptible to later YF infection [87]. This stimulated other researchers to study the possibility in Asia, because people in the dengue-endemic region of Asia might be spared of severe attack of YF. A group of Dutch workers obtained interesting but overall ambiguous results in animal tests and concluded that the effect was “relative” [34,45]. However, the interest in this hypothesis has persisted thereafter and favorable reports were published not only in Africa but in South America as well [88].

In the following discussion, the type of sequential infection is limited to the infection first by a flavivirus (excluding YFV), followed by YFV. Laboratory experiments and serologic surveys supported this hypothesis that persons who have pre-existing antibodies to mosquito-borne flaviviruses (in particular, dengue viruses (DENVs), Zika virus (ZIKV), and/or Wesselsbron virus) are better protected from subsequent YFV infection [64]. In terms of YF severity, the asymptomatic/symptomatic YF case ratio in such secondary infections was nearly 10 times higher in the persons with pre-existing antibodies to other flaviviruses, compared with the persons without [76]. More recently, it was reported that activation of heterologous CD4 T cell immunity was protective in secondary YFV infection [89]. In addition, other laboratory and field studies also supported this hypothesis [90,91,92]. This may help explain the earlier epidemiologic pattern described by Harald Frederiksen that dengue and YF outbreaks rarely occur concurrently in the same places [93]. Thus, it has been speculated that the likelihood of YF in Sri Lanka is minimal, largely based on high anti-DENV seroprevalence there [94]. This high seroprevalence observed in many other countries in tropical Asia today appears to favor this hypothesis.

However, heterotypic flavivirus antibodies do not always protect against secondary YFV infection, and protection is considered either partial, time-dependent, or serologic profile-dependent [64,95,96]. In the southeastern parts of Brazil, where YF had been absent for nearly seven decades but where large dengue outbreaks had occurred repeatedly since 1986 [97,98], a chain of severe YF outbreaks occurred in 2016–2018, resulting in 2192 cases and 753 deaths nationwide, which provided an excellent opportunity to evaluate this hypothesis. However, unfortunately serological studies on prior dengue or other flaviviral infection status of these Brazilian YF patients have not been conducted or published. 

### 5.6. Viral Interference in the Vector

Viral interference was proposed by Albert Sabin as a possible mechanism to explain the absence of YF in DEN-endemic areas [95]. This theory was supported by both in vivo and in vitro experiments. Interference of YFV replication was observed when *Ae. aegypti* were fed viremic blood of a human infected with DENV followed 2–7 days later by feeding with an artificial blood meal containing infectious YFV [95]. This result was also supported by in vitro experiments showing interference of YFV replication in mosquito cell cultures previously infected with DENV-2 [99,100]. 

More recently, a large number of insect-specific flaviviruses (commensalistic inhabitants) have been found in natural populations of mosquitoes, including *Ae. aegypti*; and their interference in arboviral replication was raised as a possibility [101]. Complicating the matter are the discoveries that fragments of insect-specific flavivirus genomes (endogenous viral elements) are found integrated in the genomes of *Aedes* mosquitoes [102]. Cell Fusing Agent Virus (CFAV), the first of these viruses described, is reported to reduce DENV and ZIKV dissemination in *Ae. aegypti* in vivo [103]. But, it is uncertain what these results mean because all subpopulations of *Ae. aegypti* in the tropics, where DENV and ZIKV transmission is widespread, have been found to be universally inhabited by CFAV. Another conflict is a report that co-infection with CFAV actually enhances DENV replication in vitro [104]. 

### 5.7. Lack of Ideal Ecologic Conditions

Two Russian scientists compared vegetation, fauna, vector, climate, human density and landscape features of the YF-enzootic regions of Africa and South America with those of tropical Asia. They concluded that the ecologic conditions ideal for YF transmission in sylvan environments had been irreversibly lost in Asia as a result of centuries of intense human activities, which did not occur at the same level of intensity in the enzootic regions of Africa or South America [105]. 

This hypothesis generally applies not to urban transmission but to sylvan transmission because, for the latter type of transmission, existence of sufficient sizes of the populations of nonhuman primates (NHPs) and of competent sylvan vectors are the minimal requirements for the persistence of YFV. Accordingly, in parts of tropical Asia where these requirements are not met, the possibility of the establishment of self-sustainable sylvan transmission is nil. This question is further explored in Section 7.5.

### 5.8. International Preventive Measures

The significance of international sanitary conventions for preventing spread of YF (Table 1) should be included as one of the possible contributing factors, even though it could have benefited Asia only after the measures were adopted. Similarly, for those who are puzzled by the total disappearance of YF epidemic in North America after the last epidemic in New Orleans in 1905 despite much weaker mosquito control in the city [106], the major reason for the cessation is better attributed to the fruit of coordinated measures to prevent YFV importation by the American states which was implemented only 3 years earlier, as YF transmission cannot persist in Louisiana without repeated viral introductions, regardless of the difference in intensity of vector control or occurrence of unusual, favorable weather in winter [107]. This is because *Ae. aegypti* does not play a role of YFV reservoir as described in Section 7.3. 

In summary, it is clear that no single hypothesis satisfactorily explains the absence of YF in Asia. Most likely, multiple preventive measures implemented over various periods of time, combined with biological and environmental factors, all contributed to preventing epidemic YFV in Asia. For example, in the 16th–19th centuries, when human movement between YF-endemic areas of Africa or Americas and Asia was negligible and the opportunity of YFV introduction almost nil because of the long journey, the hypotheses-1 and -2 likely played a critical role. When the volume of intercontinental commerce and human movement by ships or aircraft increased, interventions probably helped prevent YF dispersal. In modern times, when seroprevalence rates to DENV, Japanese encephalitis virus, and other flaviviruses sharply rose, cross-reactive flavivirus antibody and YF vaccination program may have played a more significant role. On the other hand, the hypothesis-7 could have played a role at all periods as a constant critical background. Although the principal factors involved have changed over time, interaction among multiple factors in any period explains better than any single hypothesis. Alternatively, this enigma may be understood as a result of “additive” impacts [64]. Here, the importance of holistic concept is stressed for resolving YF absence in Asia.

## 6. Routes and Directions of *Aedes aegypti* Dispersal to Asia

For studying geographic dispersal, phylogeographic methods are popularly employed. Phylogeographic analysis is most ideally applied to study geographic spread of pathogens (such as RNA viruses) that emerged recently and spread in short periods of time and for which spatio-temporally obtained specimens from all known locations are available. Other useful data include ecological factors and even intervention to control spread, if applicable [108,109]. 

When used for studying the past history of *Ae. aegypti* dispersal, however, caution is necessary because of the dependence on only one recent sampling and other factors that affect the conclusion, such as selection of DNA sources for sequencing (genome DNA, mitochondrial DNA, microsatellite, etc.), biodiversity, landscape change, climatologic dynamics over time, interaction between vectors and vertebrate hosts (including humans) [110,111,112,113] and complication derived from repeated dispersal or re-introduction to a given region, such as introgression and genetic admixing [114]. Also, even by using approximate Bayesian Computation (or ABC), a method used popularly for selection of the best scenario of dispersal history, while a group failed to identify the best scenario of *Ae. aegypti* dispersal out of Africa [115], others were successful, most likely because of differences in a combination of variables. This complicates the interpretation of phylogeographic conclusions generated among researchers.

For an outbreak of an arboviral disease transmitted by vectors to occur in a geographic area anywhere in the world, in addition to favorable climatologic and ecologic conditions, prior infestation by competent vectors and establishment of a sufficient population of susceptible host(s) are the minimal prerequisites. Accordingly, for an arbovirus transmitted by *Ae. aegypti*, prior infestation by this vector and establishment of a sufficient size of susceptible human inhabitants preferably living in urbanized environments in the tropics are the prerequisites. 

To explain the absence of YF in Asia, first it is necessary to elucidate how and when this vector dispersed there. Among multiple hypotheses proposed, it has been speculated in a dominant consensus that this mosquito evolved in Africa. According to a theory, the domesticated form of this mosquito evolved in Northern Africa after the development of the Sahara Desert around 4000–6000 years ago forced this mosquito to evolve domesticated form for survival. This population somehow migrated to sub-Saharan regions later [100,115]. According to a recent report, however, ancestral *Ae. aegypti* diverged from *Ae. mascarensis* 7 million years ago in the southwestern Indian Ocean islands and dispersed to East Africa less than 85,000 years ago, where West African lineage diverged less than 50,000 years ago, and domesticated form in Africa within the past 1,000 years [116]. Obviously, YFV was not associated with the ancient lineages or ancestral species of this mosquito, because the divergence time of East African and West African lineages of YFV based on E gene is estimated to be only 1,262 years ago [117].

Based on the branching order revealed in a phylogeographic study and using vector samples obtained in 2003–2014, it was determined that this vector first dispersed from West Africa to the New World, then from the New World to the Mediterranean region, from the Mediterranean region to Asia only after the opening of the Suez Canal in 1869, and finally from Asia to the Pacific islands or the Oceania in that order (Figure 2; red arrows) [118,119]. In this inference, an alternative scenario for direct dispersal from East Africa to Asia (Figure 2, blue arrow) was not supported statistically, despite geographic proximity. However, this East African route to Asia either directly across the Indian Ocean or via the Arabian Peninsula was once proposed [115]; and in a more recent report, this route of dispersal is inferred to have occurred by the ancestral species more than once in geologic period [120]. Although the conclusion of the latter report is interesting, it cannot be directly applied to the discussion of the dispersal of this mosquito spanning only the past several centuries because the mosquitoes in the geologic times were probably not identical to the extant *Ae. aegypti* and because of lack of reliability at most terminal nodes of the phylogenetic tree. Nevertheless, if the events at least in geologic period depicted in this report were acceptable, it would be assumed that the ancestral form of *Ae. aegypti* (or its preceding species) in the prehistoric period were originally either autogenous or hematophagous on vertebrates other than human. 

For evaluating this theory of great interest [119], the crucially-important report used as a collaborative evidence to support the theory [121] needs to be re-examined. In this report eastward movement of a pandemic of a dengue-like disease outbreak across tropical Asia was documented. The pandemic began in the region stretching from Zanzibar to Tihama region of the Arabian Peninsula including Yemen in 1870, dispersed to India (in 1871–1872) and to more Asian countries as far east as Hong Kong (in 1872), before reaching Queensland of Australia through a different route via Mauritius in 1873 [122,123]. At that time, the opening of the Canal provided opportunities for large numbers of Europeans to migrate to Asia in search of economic gains and other opportunities. The major problem is that this sequence of epidemiologic events was interpreted to represent eastward dispersal of the vector [121]. 

The peculiar feature of this pandemic is that in 1872 alone the outbreaks were reported from multiple countries in tropical Asia almost simultaneously. It is emphasized that the possibility of introduction of vectors to a virgin territory previously free of them and of instantaneous eruption of a pandemic covering multiple countries by an arbovirus is almost nil, because it takes at least a few years for vectors to establish a sufficient level of infestation in any new territory for vectors to be able to cause an outbreak. Thus, unlike the dispersal patterns of directly-transmitted viral diseases of humans in new territories, such as COVID-19 coronavirus, influenza and measles, for an outbreak of an arboviral disease to occur in a virgin territory, a prior establishment of vectors is a prerequisite. This has been shown by the recent dispersals of arboviruses of the Old World into new territories where the prior infestation of vectors had been well known before dispersal (such as chikungunya virus [CHIKV] and ZIKV in the New World) or unknown (such as WNV in the Americas and Usutu virus in Europe). Accordingly, this eastward movement is better interpreted instead as the movement of the etiologic agent of the dengue-like illness in association with human movement across the Asian/Pacific countries that had been already infested by *Ae. aegypti* [124]. 

One of the other weaknesses of this hypothesis is that the dispersal between the Americas and Asia via the Mediterranean is hypothetical, because mosquito specimens from the Mediterranean area were unavailable. This vector, which had once infested the Mediterranean and the Black Sea regions [125,126], mysteriously disappeared from both regions completely after early 1940s or 1950s [110,127]. Either installation of piped water system or application of DDT has been speculated to be the reason. However, the fact that 13 countries in the Mediterranean and Black Sea regions signed an agreement to control *Ae. aegypti* in 1935 cannot be overlooked [127].

Although a recent *Ae. aegypti* specimen from the Black Sea region is speculated to fill the gap in the dispersal route of this hypothesis [128], whether or not the recent specimen is a direct descendant of the “presumably extinct” earlier Mediterranean population is uncertain, because re-infestation in the Black Sea areas was first reported only since 2000 [129,130,131]. 

Actually, as to the date of this vector establishment in Asia, well before 1800 is more probable, because numerous outbreaks of DEN-like or chikungunya (CHIK)-like illnesses in urban areas were recorded in Asia since the 18th century [132]. In fact, the true etiologic agent of the outbreak in Batavia (currently Jakarta), Indonesia in 1779–1780 [133] is now considered almost certainly chikungunya virus (CHIKV) [134]. Since CHIKV is a virus of African origin principally transmitted by *Ae. aegypti*, early records of its introduction to Asia from Africa better explains this historical account. Other records show that an earlier outbreak of a severe joint syndrome, which is again considered almost certainly CHIK [134] and which started in Zanzibar of Tanzania in 1823, just like the aforementioned dengue-like disease outbreak of 1870, spread to India causing outbreaks in 3 separate locations in 1824–1825 [122]. This strongly suggested that *Ae. aegypti* dispersed from East Africa to Asia (Figure 2, blue arrow) multiple times before 1860s. This occurred again more recently, as shown in the CHIKV involved in the 2005 pandemic [135]. This is another reason why the validity of the Mediterranean route of vector dispersal was questioned [136]. The facts that do not favor the possibility of East Africa-Asia dispersal have been historical scarcity of YF outbreak in East Africa, general absence of domesticated form of the vector, and its insufficient transmissibility of the vector [110]. However, in the past, sufficient attention has not been paid to the aforementioned multiple records of the early outbreaks of CHIK-like illness in Asia. Furthermore, at least in Rabai, Kenya, domesticated form of *Ae. aegypti* has been known to coexist with sylvan form for many years [137].

On the other hand, another alternative argument that instead it was actually *Ae. albopictus* (a rural or periurban species indigenous to Asia) that played the role in the old CHIKV transmission in Asia [138] is weak, because the efficacy of CHIKV transmission of this Asian mosquito evolved for the first time in 2005 only after viral mutation made it possible [139]. In the current CHIK transmission in the Americas too, the major vector involved is again *Ae. aegypti,* despite co-infestation with *Ae. albopictus* in the affected regions. Thus, the possibility of CHIK epidemic by *Ae. albopictus* in Asia before 1900 is highly unlikely. 

Theoretically, vector dispersal from West/Central Africa to North Africa along the overland slave trade (Figure 2, orange arrow) is also possible. The tendency of domesticated *Ae. aegypti* to pursue humans (in particular, those carrying personal belonging or heavy tools for work) who walk slowly and stop frequently has been observed in the episodes of this mosquito pursuing caravans of pilgrims trekking towards sacred shrines or temples, *Ae. aegypti* infestation of underground shafts or wells in gold mines in an arid part of Australia, and in house-to-house movement in urban area of Asia [140,141]. In fact, a large outbreak of severe arthritis resembling CHIK was recorded in Egypt in 1658 [142]. Thus, a possibility of further dispersal from Egypt to Asia was also once considered [115]. Accordingly, an alternative possibility of early dispersal of *Ae. aegypti* infected by CHIKV dispersing from somewhere in Africa to Asia (Figure 2, blue or orange arrow) independently of the proposed route from the Mediterranean region [119] cannot be entirely dismissed. Then, it is possible that this early *Ae. aegypti* population from Africa subsequently became extinct or was displaced by other lineage later. 

Another interest concerns the history of introduction of mosquitoes into Hawaii, where the earliest introduction of mosquitoes was recorded with intense curiosity by entomologists and naturalists, because they had never seen mosquitoes there until 1826 when *Culex* mosquitoes were accidentally introduced. Although not all agree on the exact date of the introduction of *Ae. aegypti* to Hawaii, a short span of period (1828–1830) well before the opening of Suez Canal is supported by the majority [20,143,144]. It is also believed in Hawaii that while *Ae. aegypti* arrived earlier from the East (probably implying the Americas; Figure 2, green arrow), *Ae. albopictus* arrived much later from the West (presumably either Western Pacific or Asia), both by ships [144]. Although the possibility of vector dispersal from the Pacific coast of the Americas to Asia by the Manila Galleon trade across the Pacific between 1565 and 1815 [145] cannot be totally dismissed at present, the earliest date of the arrival of *Ae. aegypti* in Hawaii is clearly unrelated to this trade. A study based on DNA sequence and nucleotide polymorphism analysis also presented a possibility that this vector dispersed westward from the Americas to Asia and Australia [110]. For the dispersal of *Ae. aegypti* to the Pacific, while the phylogeographic studies based on recent sampling [118,119] supports eastward dispersal of the vector from the Americas via the Suez Canal (Figure 2, red arrows), westward introductions of both YF and *Ae. aegypti* to the Pacific from the Americas occurred at least once (Figure 2, green arrow) [19,144].

In addition to extinction, a possibility of displacement by a more dominant vector lineage that arrived later also must be considered. If either one actually happened, naturally the phylogenetically inferred direction of geographic dispersal of this mosquito based on recent sampling does not reveal such earlier events in the nearly five centuries of dispersal history, because of unavailability of the early specimens, even though studies using vector specimens obtained recently may be highly useful for understanding the genetic relationship among extant geographic strains. Thus, the limitation of phylogeographic studies based only on recent one-time sampling to infer the long history needs to be considered, particularly for the organisms for which human activities played a significant role in addition to natural mode of dispersal.

The aforementioned discussion stresses that when inferring the past history of the geographic dispersal of a vector-borne viral disease, integration of epidemiologic, ecological and entomologic histories as well as other biological sources of information and human activities as collaborative data is important for improving the accuracy of phylogeographic conclusion. Sometimes, what matters is quality rather than number of the documents supporting a particular scenario, when comparing proposed hypotheses. As an example, when the theory of New World origin of YF largely based on numerous records of YF (or YF-like) outbreaks in the Americas beginning in 1648 in Yucatán, Mexico was supported by most scientists and physicians, Carter placed a greater importance on the quality of a few historical documents on a similar disease reported from Africa prior to 1600. Then he proposed a theory of African origin [11] ahead of the recognition of the important role slave trade played as a mechanism of YF dispersal from Africa to the Americas [146] and well before revelation of molecular phylogenetic evidence obtained on the vector or virus [147].

## 7. Possibility of the Establishment of Transmission Cycle in Asia

Because of the prohibition of the possession and use of live YFV in such countries as India, Singapore, Malaysia, Philippines and Indonesia, research to explore this possibility was not conducted before WWII in Asian tropics, with the only exception of vaccine production in India as described earlier. Also, as described, dried mosquito eggs of Asian strains of *Ae. aegypti* were mailed to Europe and Asian monkeys were used in the laboratories there. Furthermore, YFV vector competence and monkey susceptibility were also studied there [44,45,148,149,150]. In the early 1960s, YF vaccination was contemplated in Malaysia, but a preliminary study was limited to evaluation of the impact of pre-existing cross-reactive antiflaviviral antibodies upon vaccination with 17D vaccine [151].

When the possibility of YF in Asia is the topic of discussion, because such a generalized question itself is so ambiguous that it may refer to either mere imported case(s), autochthonous urban transmission, sylvan transmission or the combination of second and third modes of transmission. In this section, imported case(s) is excluded. The following exercise is designed for us to be well-prepared in advance, should an unfortunate event of YF outbreak ever occur in Asia, by reviewing available data on the patterns of transmission and other knowledge gained in Africa and South America [64,152,153]. Based on the pattern in Africa, it is assumed that interface transmission evolves only after a firm establishment of sylvan transmission. Because establishment of sylvan transmission in Asia itself is questionable, the possibility of interface transmission is not discussed. 

**Figure 3:**
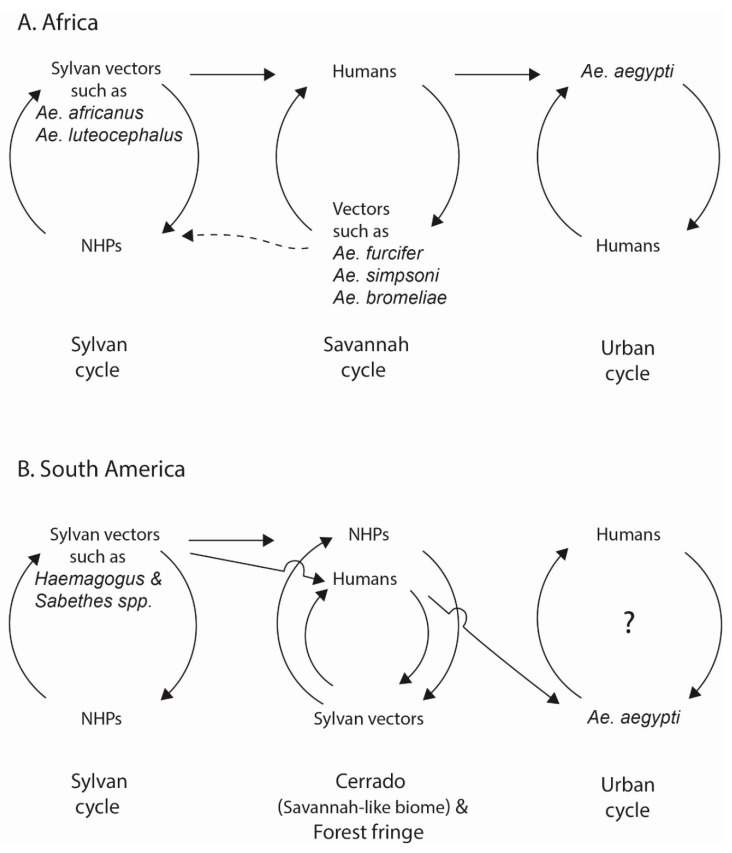
Patterns of YF transmission in Africa and South America (Vertical transmission in the vectors is not shown because it is infrequent).

Figure 3A illustrates the three patterns of transmission recognized in Africa, revealing transition from sylvan transmission to interface transmission (savanna cycle) in the “zone of emergence” [154], and then to urban transmission. Strictly speaking, the boundary of the vast zone of sylvan transmission is ambiguous and shifts dynamically over time. Similarly, classification of environments to three categories is arbitrary, due to the complexity of human activities and variation of the spatial pattern of clustering of houses, depending on location. Interface and urban transmission patterns are also called “wet savannah” and “dry savannah/urban” cycles, respectively. Thus, depending on location, *Ae. africanus* plays a role in both sylvan and interface; and *Ae. aegypti* and *Ae. simpsoni* may be exophic (zoophilic) or endophilic (anthropophilic). Also, *Ae. aegypti* in parts of Ivory Coast is exceptionally a nocturnal feeder.

In South America, traditionally only two patterns have been recognized, sylvan and urban. The dynamic shift of the boundary of sylvan transmission in South America was once characterized as “wandering” or compared to protrusion and retraction of the pseudopod of amoeba by Richard M. Taylor. The pattern of interface transmission, which is similar to but different from that in Africa is shown in Figure 3B. Selected topics of these patterns of transmission learned from South America are commented on first to assist the discussion of their possibility in Asia which follows.

### 7.1. Urban Transmission in South America and Trinidad

The distinction between “urban” and “rural” in the context of arbovirus transmission has been an unresolved issue [155]. In the Americas, because classification as “urban transmission” has been ambiguous often for lack of evidence of the involvement of *Ae. aegypti*, it is important to investigate if confirmed “urban” cases are merely urban residents infected in rural areas, the results of authentic urban transmission by *Ae. aegypti*, or the extension of interface transmission [156]. This is illustrated in an “urban outbreak” in Sena Madureira in the state of Acre of Brazil in 1942 [157], Santa Cruz, Bolivia in 1998 [158], and San Lorenzo, Paraguay in 2008 [159], all involving less than several “urban” cases. In a much larger outbreak in Sena Madureira in 1937–1938, for example, the numbers of “urban” and “rural” patients were 3 and 473, respectively [160]. Although the 3 cases were called “urban” simply because urban residents contracted YF without a history of travel to rural area, evidence of secondary transmission by *Ae. aegypti* was not documented. The other possibility must be considered, because sylvan vectors in South America, such as *Haemagogus* spp., generally known as canopy feeders in the forest, are not only known to travel long distances but diurnally bite residents indoors in the urban houses bordering forest or interface [161,162]. 

In Brazil, often urban transmission of YF is reported in relation to particular municipalities. For those unfamiliar with the system of administrative subdivision of the country, a caution needs to be exercised. A “municipality” in Brazil is, unlike in other countries where it is almost synonymous with a city occupying a limited space, a subdivision within a state occupying a far larger area. As an example, the sparsely-populated municipality of Sena Madureira (of Acre state) occupies 9760 sq. miles much of which used to be a vast wilderness. The municipality of São Paulo (of São Paulo state) is much smaller, but huge megacities within are not too far from nature reserves (fragments of the Atlantic Forest) where sylvan YF vectors and NHPs are abundant, facilitating increased contact of urban residents with YFV [97,163]. 

Accordingly, in this review, most outbreaks in South America and Trinidad after the last true large-scale urban outbreak in Rio de Janeiro state in 1928–1929 [164,165,166,167] are classified as “sporadic outbreaks” of uncertain urban status [156]. After 1929, true urban transmission by *Ae. aegypti* verified by virus isolation was recorded in one case during an outbreak in Trinidad in 1954 [81]. For these reasons, the practice of characterizing the post-1930s outbreaks in the Americas as “urban” has been questioned [168] (Figure 3B). 

Another issue that has not been clarified sufficiently even in Africa relates to the evidence of YFV persistence in urban areas. Here, it is important to recognize a significant difference among the viruses transmitted by *Ae. aegypti* regarding the duration of persistence in urban areas. For the four serotypes of DENV, the probability of the establishment of viral persistence in a fixed, urban area for several years or longer increases when human population size exceeds a minimal population size, even when seroprevalence of homologous or cross-reactive antibodies is still considerable [169,170]. However, CHIKV and ZIKV disappear quickly after causing an extensive outbreak in urban areas even when large proportions of populations still remain susceptible but reappear in other locations unpredictably, jumping from one location to another [171,172,173]. This pattern has been repeated not only in Africa and Asia but in the Americas [174,175]. Furthermore, the duration of YF outbreak in a given urban area is, like CHIKV and ZIKV, short. Currently, a 3-year persistence of YFV in an interface area is considered exceptionally long in Brazil [163].

Thus, by arbitrarily assuming that the impacts of multiple factors, such as possibility of re-introduction of the same virus and quality of vector control activity be equal among these viruses, the difference in the duration of viral persistence in urban areas between DENV on the one hand and YFV/CHIKV/ZIKV on the other is striking. Here lies the need of further research because of enormous importance in epidemiology. 

### 7.2. Interface Transmission in South America

A unique pattern similar to but different from the pattern in Africa was recognized since 1934–1940 and more recently in the 2016–2018 outbreaks in the vast area of Brazil covering several states including Mato Grosso, Goiás, Minas Gerais, Sao Paulo and others [97,152,176] (Figure 3B). This pattern was interpreted by Fred L. Soper as a byproduct of sylvan transmission spilling into urbanized areas in the interface or even to urban environments, which is characterized by the involvement of sylvan vectors (but not *Ae. aegypti*), causing a combination of epizootic in NHPs and epidemic [152,156]. The major part of this interface in Brazil covers not only the vast central high plateau (“Cerrado” or “planalto”) known for its savannah-like biome but adjacent areas including forest fringes. One notable feature of this epidemic/epizootic is rapid mobility of foci and long distance foci travel over a relatively short period. In the 1934–1940 epidemic/epizootic, the foci travelled at an estimated rate of 200 km per month at its peak for a total length of 1500 km [139]. In the outbreak in Central America (1949–1957), foci travelled 1550 miles from Panama to the Guatemala-Mexico border at approximately 0.5 miles/day. In the 2008–2009 epizootics, foci travelled 182 km/year [177], while in the 2016–2018 epidemic/epizootic, the mobility was 3 km/day for a distance of 600 km. Although human activities (including travel) contribute to the high mobility, considerable flight range of sylvan vectors (11.5 km/release or 3.5–5.0 km/day for *Haemagogus janthinomys* and 5.7 km/release for *Hg. leucocelaenus*) [161,163] is another factor, even though flight distance may be affected by wind. This is compared with movement for 3 km in 4–8 days by *Ae. africanus* in Africa [64], while foci traveled 500 km in a few months [77]. Patas monkeys are known to forage across savanna as far as 16 km/day; and the area foraged by bands of these monkeys is as large as 300 km^2^ [64]. While outdoor activities and movement of the residents contribute to human infections, the importance of diurnal, multiple and indoor feeding activity of these sylvan vectors in the houses in the interface area adjacent to the habitats of NHPs has been recognized in multiple countries in South America [98,162].

### 7.3. Spillback in South America- A Hypothesis

Although how the sylvan cycle of YFV evolved in South America has not been clearly elucidated yet, the mechanism involved must have been spillback [4]. Because this is an important subject to discuss regarding YF possibility in Asia, a hypothesis is presented, based on the data accumulated in Brazil [178]. The two concepts underlying this hypothesis are as follows:

First, for a spillback to occur, the minimal requirements that must be met are occurrence of competent primatophilic sylvan vectors, sufficient sizes of NHP populations, and “optimal corridor.” In this review, “optimal corridor” refers to an interface zone separating sylvan area from urban area, which is characterized by the ecologic conditions and human activities conducive to contact either between sylvan vector and YFV-infected humans within urban areas (for spillback) or between virus-infected sylvan vectors and humans in interface and/or urban environments (for spillover). The shorter the distance between sylvan and urban environments is, higher is the probability of the occurrence of the contact between YFV and human. Although rapid transportation in modern periods shortened the time to travel the optimal corridor, it facilitated spillover but not spillback, because physical distance did not become shorter for the sylvan vectors to travel. The favorable human activities include frequent transport of agricultural and forestry commodities and associated business, logging, drilling, construction, mining, hunting and recreational activities. (In modern periods, recreational activities include ecotourism) 

Thus, if the distance is very short and human activities favorable, the probabilities of both spillover and spillback would be increased. When the distance is long but human activities favorable, while spillback does not occur, at least spillover can happen occasionally. When the distance is long and human activities unfavorable, neither spillover nor spillback would occur. 

For the estimation of the distance for an optimal YFV-human contact, the average distance of 1.7 km between residences of confirmed YF cases and the nearest site of YFV-infected NHPs, compared with 39.1 km for the mean minimal distance between residences of non-infected persons and the nearest location of infected NHPs, which were determined during a recent outbreak [176], is useful as a rough guide to infer the distance in the nascent period of urban development when spillback presumably took place. In the future, quantification of important human activities in the interface in the development of spillover would improve mathematical modeling in epidemiologic analyses of YF, using a variety of concepts developed elsewhere [179]. In fact, daily commuting pattern and means of transportation linking communities, human density, vector suitability and others have been parameterized in a study [180]. 

Second, the unique biological traits of the major sylvan vectors of the Americas, in particular *Haemagogus* and *Sabethes* mosquitoes, are of critical importance in this hypothesis. As described earlier, those vectors travel long distances and engage in blood feeding indoors in daytime in the houses located close to interface area [161,162]. Furthermore, *Haemagogus* and *Sabethes* mosquitoes are, like *Ae. aegypti*, diurnal, multiple feeders, traits considered more efficient in virus transmission [181]. These traits increase contact between the sylvan vectors and YFV-infected humans in urban environments where *Ae. aegypti* plays a dominant role in urban transmission. This facilitates transfer of YFV to sylvan vectors (spillback). 

According to a molecular clock study, the mean estimated date of the most common ancestor of YFV in the Americas is estimated to be 1822 [177], while the earliest outbreak recorded in Brazil occurred in 1640 [178]. The following hypothesized scenario is based on an epidemiologic account.

The sequence of the hypothesized events related to the evolution of spillback with an emphasis on Brazilian setting is as follows:

Stage 1: In the early period of history, when repeated introductions of YFV to South America occurred in association with slave trade, urban areas were still small and economic development was limited, which allowed sylvan environments in the hinterland surrounding the urban areas still undisturbed. Accordingly, the distance between sylvan and urban environments was short, which provided frequent opportunities for the sylvan vectors to acquire YFV during their diurnal feeding activity when urban transmission of YFV by *Ae. aegypti* was ongoing. Because this virus transfer to sylvan vectors occurred directly in the shared urban environment, involvement of bridge vectors was unnecessary. Currently, a gradual spillback process from urban to sylvan environment by means of bridge vectors is popularly considered [182], while its occurrence directly in sylvan environments has also been proposed [183].

Stage 2: After countless numbers of virus transfer to sylvan vectors (spillback) over decades or much longer, eventually a sylvan cycle of YFV transmission was firmly established as a cycle independent of urban cycle. It has been speculated by some that sylvan cycle evolved either around 1907 when Roberto Franco raised a suspicion during an outbreak in Muzo in Colombia (known for the emerald mines) or in 1932 when Fred L. Soper definitively confirmed it in the state of Espirito Santo in Brazil.

Although the earliest observation of “jungle yellow fever” was actually made in Muzo of Colombia in 1885 [160], in this hypothesis it is assumed that sylvan transmission cycle had been established much earlier most likely in multiple locations along the Andes in South America. 

Thereafter, the two sources of YFV for urban transmission were the virus introduced to urban areas through the “optimal corridor” as a result of spillover of sylvan cycle of transmission (first source) and imported virus from anywhere in the Americas infested by YFV and from Africa (second source). Accordingly, in contrast to the areas in South America where both sources are involved in an endemic/enzootic transmission, in other areas where transmission depended on the second source only, occurrence of YF outbreak was mostly sporadic. When “seemingly continual” YF cases or outbreaks occurred in the latter locations for a few years or several years consecutively, as they occurred often in parts of North America (such as the consecutive yearly outbreaks in 1791–1800 in Philadelphia), Central America, the Caribbean and even in Gibraltar, actually they were mostly the consequences of repeated virus introductions, except for a few unusually prolonged dispersal such as that observed in Central America in 1949–1957. 

It is necessary to emphasize that currently there exists no evidence of YFV establishing an “endemic” (or perpetual) transmission in urban environment. Accordingly, the possibility of urban “endemicity” as the third source of virus for either spillback or spillover is ruled out. This is supported by the phylogenetic studies. Consistent isolation of genotype- and site-specific DENV-1 over 13 years has been considered an evidence of viral persistence [184]. While DENV isolates from epidemics in urban areas are separated at the roots of phylogenetic trees from sylvan strains [185,186], YFV strains isolated from epidemics or epizootics are not segregated similarly, suggesting that the strains involved in spillover events in the interface or urban residents are all direct descendants of sylvan lineages (first source) [176,177]. The very low mutation rate in YFV also supports that this virus has not adapted from sylvan to urban environment yet. Furthermore, occurrence of vertical transmission of YFV in *Ae. aegypti* under natural conditions is extremely rare. Actually, after its first discovery made in 1903 by Émile Marchoux and Paul-Louis Simond [187], it took more than 90 years to confirm it [188], despite numerous attempts to prove. In other words, *Ae. aegypti* does not play a role of reservoir of YFV not only in the Americas but in Africa.

Stage 3: As urban areas grew and economic activities expanded in the hinterland, the distance grew longer. Thus, the opportunity of YFV (of the first source)-human contact declined. As a result, the probability of the occurrence of spillover was reduced. As for the second source of virus, it dried up totally as the result of the combination of human activities and interventions, such as abolition of slavery (around 1888), implementation of YF prevention strategies illustrated by the Oswaldo Cruz’s vector control program in Rio de Janeiro beginning 1905 followed by another along the Amazon River basin in 1910-1913, vaccination program (since 1938), and more centralized *Ae. aegypti* control campaigns (beginning 1929 by the national government, the RF, and PAHO) [156]). On top of these activities, there was a campaign to eradicate this vector in the Americas by the PAHO between 1947 and early 1970s. This is reflected in the sharp proportional decline of urban YF cases but proportional increase in rural (interface) transmission observed in a fixed location in Brazil continuously monitored in the 1930s [160]. At any rate, roughly after early 1930s, urban transmission in Brazil (and elsewhere in the Americas) has become spillover-dependent. Since spillover occurs at irregular intervals depending on the population sizes of NHPs and other unknown factors, the occurrence of urban transmission has become infrequent. This explains why true examples of urban transmission there have become rare in modern periods, most likely without a significant change (if any) in vector competence.

### 7.4. Urban Transmission in Asia

In the age of accelerated international travel, the probability of transmission of limited duration in urban areas of tropical Asia is always high, given intensive infestation of the vector, huge sizes of human population, and total lack of immunity, since any screening method (such as thermal monitoring at international airports) is never 100% fail-proof [189]. Furthermore, some certificates of YF vaccination presented by arriving international travelers may not be authentic, and inspection of certificates is inadequate in some countries. Distinguishing autochthonous urban transmission from imported cases is important. If urban transmission happens, the size of outbreak is expected to vary greatly among countries, depending on the level of preparedness, quality of communication system, resources available for emergency measures, speed of implementation and efficacy of emergency control measures taken by local and national authorities, the number of people exposed to infected mosquitoes at initial stage, and the level of citizen cooperation in the execution of control program. As human migration across national border (legal and illegal) has increased recently worldwide [190], surveillance of the health of migrants has become another necessity for YF prevention.

For the Asian strains of *Ae. aegypti*, their susceptibility to YFV was confirmed in multiple laboratories [44,45,82,148,150]. As for transmission in periurban environments, the vectorial potential of *Ae. albopictus* has been found variable in the past, but recent studies confirmed more frequently the efficacy [44,45,191,192,193]. *Ae. malayensis*, another peridomestic mosquito in Singapore, was found to be also competent for YFV at least under laboratory conditions [194]. However, for all potential vectors in Asia, vertical transmission should be studied. 

One unique environmental factor in Asia is ubiquitous co-habitation of monkeys with humans in many urbanized areas including tourist spots. High adaptability of some NHPs in Asia to diverse environments renders their classification into urban or sylvan species difficult. As examples, some species, such as *M. mulatta* (rhesus macaque) and *M. sinica* (toque macaque), occur not only in lowland but in highland, while other monkeys occur in both wet and dry areas. Because vectors associated with a given species of macaque would likely be different at different habitats, for a given susceptible macaque, its potential role in YFV transmission must be ideally studied in the vectors in multiple habitats (such as urban and sylvan). If a given NHP is preferred by the vectors in more than one environment, a possibility of a unique pattern of YFV transmission not known either in Africa or South America might be revealed.

Although high frequency of *Ae. aegypti* feeding on both human and rhesus macaque is known [195], the paucity of local studies on the role of those urban monkeys in arboviral transmission in Asia has been a problem. The susceptibilities of *M. mulatta* and *M. fascicularis* (cynomolgus macaque) to YFV are known [196,197]. Interestingly, while the mortality in adult cynomolgus macaque was low [198], it was high in young age groups. Also, while this species in Malaysia was resistant, the same species in India was susceptible [44,45]. Also, lower susceptibility of the lowland population of *M. sinica* in India was confirmed [34,60]. However, more laboratory research is necessary to examine the macaque’s possible role in urban YF transmission. If confirmed, the strategies of YF control need to be modified accordingly.

### 7.5. Sylvan (Enzootic) Transmission in Asia

Whether or not sylvan transmission cycle of YFV will be established in Asian environments when YF outbreak occurs is a far more difficult question, since currently there exists no consensus over exactly how YFV established a sylvan transmission in South America by spillback in the first place. When discussing the possibility of a firm establishment of sylvan transmission of YFV in Asia, it is important to recognize once again the significant difference among the viruses transmitted by *Ae. aegypti*. With exceptions of the 4 serotypes of DENV, predicting the possibility of permanent establishment of YFV, CHIKV, and ZIKV in new territories is far more difficult. As an example, if the etiologic agent of the 1828 pandemic of severe arthralgia that prevailed in the Americas was CHIKV, as strongly speculated [134,199,200], the lineage must have become extinct. Furthermore, like CHIKV, the exact boundaries of sylvan transmission of ZIKV in Africa and Asia have never been clearly defined. Accordingly, it is still premature to predict if the current lineages of CHIKV and ZIKV in the Americas will firmly establish a sylvan cycle, given the rapid decline of transmission of ZIKV [174,175] and similar decline of CHIKV, with an exception in Brazil [201]. Even if the aforementioned spillback hypothesis is not acceptable, identification of Asian sylvan vectors comparable in transmission efficacy to *Haemagogus* and *Sabethes* mosquitoes of New World must be a first priority of research, because they do not occur in Asia. If any one of the aforementioned three minimal requirements (competent primatophilic sylvan vectors, susceptible NHPs, and “optimal corridor”) is missing, sylvan transmission of YFV will not evolve in Asia. If so, the modified version of the hypothesis-7 (Section 5) at least partially explains the YF absence in Asia.

It is also assumed that there exists no possibility of Asian vertebrates other than NHPs serving as hosts for YFV, since such vertebrates have never been found to play a major role in transmission in Africa or in the Americas.

In India, Payyalore K.I. Rajagopalan initiated an ecosystem project to assess the possibility of YF transmission at Devimane Ghat in Karnataka State, an evergreen tropical forest. But, the project was abruptly cancelled because of an unexpected national emergency [202]. Accordingly, we know practically nothing about possible sylvan vectors and vertebrate hosts of YFV in Asia. Peter F. Mattingly wondered if Asian mosquitoes belonging to genus *Heizmannia* might play a role of sylvan YFV vectors comparable to that of *Ae. africanus* in Africa and *Haemagogus* spp. in the Americas. However, his speculation was solely based on taxonomic affiliation but not on vector competence [203]. A recent phylogenetic study revealed that *Haemagogus* and *Heizmannia* mosquitoes are not close relatives [204].

Identification of the potential diurnal sylvan primatophilic vectors of YFV in Asia is critically important, because vectors (but not vertebrates) are the true reservoirs of RNA arboviruses including YFV, based on the current reservoir definition [205]. Because of the paucity of sylvan mosquito study in Asia, the findings of two studies (one by Albert Rudnick for sylvan DENV transmission in Peninsular Malaysia and another in rural areas of Sarawak) may or may not provide useful information for YFV [206,207]. In India, *Ae. vittatus* and *Ae. scutellaris* were found susceptible to YFV [60]. Although drought-resistant *Ae. vittatus* is only a minor vector of YFV in Africa, its vertical transmission in an Asian strain was not found to be inferior [208]. However, the other serious problem is that little has been done to learn if those vectors in Asia tested for YFV vector competence are actually primatophilic.

Regarding Asian non-human primates (NHPs), their arbovirus association has been confirmed by virus isolation or serologic tests for DENV and other viruses in the past. They are *Macaca philippinensis* of the Philippines; *M. nemestrina* (pig-tail macaque), *Presbytis cristana*, *P. melalophos*, and *P. obscura* of Malaysia; and *Macaca sinica* of Sri Lanka. Currently little is known about the potential role of those sylvan NHPs as hosts of YFV in transmission. Indonesia is one of four countries in the world known for the rich fauna of NHPs. Although NHP populations have been reduced considerably due to a combination of expansion of agriculture, commercial activities, inadequate conservation, and culling to control nuisance problem for urban residents and other reasons, the concern still exists.

## 8. Other Enigmas

Early in the 1930s, when *Ae. aegypti* as the vector of YFV was firmly established, occasional observations of YF patients in South America in the absence of this vector was one of the early enigmas. Although that enigma was quickly resolved by the discovery of sylvan transmission, as research progressed further, it ironically generated new enigmas, some of which have remained without a clear resolution. Selected topics that have largely remained problematic or that require further clarification are discussed.

### 8.1. Viral Maintenance in Vectors

#### 8.1.1. Vertical Transmission (VT) of Flaviviruses in Vector Mosquitoes

Vertical transmission (VT) of flaviviruses in mosquito vectors has been long thought to be a mechanism by which these viruses survive under adverse environmental conditions [209]. Although VT consists of transovarial transmission and transovum transmission, most studies have focused on the former, since it is more difficult to confirm the latter. Sexual transmission as another form of VT is an unresolved issue.

As mentioned earlier (Section 7.3), it took more than 90 years to confirm occurrence of VT of YFV in *Ae. aegypti* under natural conditions, since its first recognition. In the meanwhile, the virus was sporadically isolated from *Ae. furcifer-taylori* males in Africa [210] and from nulliparous *Haemagogus janthinomys* females and *Hg. leucocelaenus* in Brazil [211,212]. The conclusions of many studies and reviews, while acknowledging occurrence of VT in nature, have been that this mode of transmission is mathematically insufficiently robust to sustain virus and, thus, epidemiologically insignificant or that its role in virus maintenance is questionable [209,213,214].

On the other hand, others have argued that VT is not necessarily a fixed character but changes dynamically over time within and between vector generations and that in too many studies only the detection of virus in the first gonotrophic cycle was emphasized, while subsequent cycles (in which virus positivity rate is expected to rise) were not [215]. Another problem peculiar to YFV is the fact that sylvan vectors (rather than *Ae. aegypti*) should be studied but accurate identification of the sites for an optimal sampling for evidence of VT is extremely difficult, because the boundary of sylvan or interface transmission is ambiguous and changes constantly. Because of this problem, the number of attempts to detect VT under natural conditions has been very small, which further enhanced negative impression. Also, not all vectors have been investigated in depth in longitudinal studies.

#### 8.1.2. Larval Transmission

That many arboviruses can be transmitted horizontally in aquatic conditions between larvae of the vectors was actually revealed for the first time for YFV in 1938 by Loring Whitman and Paulo C.A. Antunes in Brazil [216]. In nature, this mode of transmission is expected to occur most often when infected larvae are cannibalized and release virus. Thereafter, this mode of transmission was confirmed for other arboviruses in laboratory experiments [217], but few field investigations have been conducted.

Although the importance of this mode of transmission has been discounted because the larva at the beginning of this chain of transmission is itself a product of VT (a rare event) [214], because many mosquito larvae breed in congregation in the same aquatic environment, theoretically this would be an efficient mechanism to infect a large number of larvae simultaneously. At any rate, more field studies are necessary to evaluate this mechanism.

#### 8.1.3. Expectoration

Because female mosquitoes need sources of protein, usually only blood feeding activity has been emphasized for the role of females in arbovirology. As a result, the importance of nectar feeding for energy source has been traditionally recognized for the survival of males. However, recent studies revealed that this feeding is also common in females as well [218,219]. As an example, female *Ae*. *aegypti* outdoors were found to engage in nectar feeding far more frequently than it was thought before. In this mosquito, while stylet is used for blood detection, labrum is used for detecting nectar [220]. This opened a new approach to vector control using a toxic honey bait in Africa [221]. Furthermore, the discovery that infected females engaging in nectar feeding expectorate arboviruses into nectar was found to be useful for an arbovirus surveillance [222]. In expectoration, saliva containing arbovirus is discharged when females engage in feeding activities. Accordingly, if the same nectar is shared by multiple males and females, theoretically virus transmission occurs between mosquitoes. Thus, like larval transmission above, this needs to be investigated further.

#### 8.1.4. Sufficient Populations of Infected Vectors and Year-Long Activity of Vectors

The idea based on sufficient numbers of infected vectors either to survive dry season to re-introduce virus at the onset of rainy season or persistent survival during adverse climate has been one of the traditional explanations for the YF transmission in Africa [64]. The key to this proposal was total number of infected vectors belonging to multiple genera and high reproductive rates of those vectors which compensate the low proportion of infected mosquitoes in a given location.

According to a traditional concept, because activity of vectors in the tropics ceases during a period of adverse climate, there must be a special mechanism for virus maintenance. In Central America, Pedro Galindo emphasized the potential role of long-lived, drought-resistant adult *Sabethes chloropterus* [223]. However, later studies showed year-long vector activity, even though activity dropped sharply during the adverse period. Accordingly, in such environments, no special mechanism of viral maintenance was necessary. In Trinidad, breeding of *Hg. janthinomys* through four seasons was conclusively confirmed by Elisha S. Tikasingh [224]. In eastern states of Brazil, persistent activity of at least *Hg. leucocelaenus* and possibly of *Hg. janthinomys* during dry season in the forest fragments was observed [163,225]; and a long-term study revealed that YFV persisted in the same forest area (in the interface environment) for at least 3 years consecutively [226].

If any of the above mechanisms does not provide a fully satisfactory explanation singularly in all circumstances, combinations of those mechanisms varying in the composition of mechanisms, mosquito fauna, climatologic factor, and location may better explain virus persistence in vectors.

### 8.2. Dead-End Hosts, Incidental Hosts, Survival of Vectors, Dilution Effect, and Immune Hosts

The term “dead-end host” was originally coined to refer to the vertebrate hosts which are infected but which cannot serve as a source of virus for further transmission, because, if they survive infection, they will become immune (immune host) [152]. Lately, “incidental host” has been used almost synonymously with “dead-end host,” though it has never been officially used by the WHO. Thus, conceptual ambiguity is a shared problem among those topics.

In this review, “dead-end host” is used for a host of primary importance in biological transmission which survives infection and develops immunity to a particular virus, while “incidental host” is used to refer to any host of secondary importance regardless of immune status. The reason for this segregation is as follows. First, we assume that YFV transmission between vectors is insufficient for viral maintenance and only vertebrate hosts matter for the survival of virus. Then, if YF seroprevalence in NHPs (primary hosts) is as high as 95%, as in sylvan transmission of YF [64], viral maintenance is difficult, unless newborn NHPs replenish supply of susceptible hosts very quickly. In fact, this was used to argue that in the total disappearance of susceptible hosts, YFV would become extinct [227]. It has not happened, however. According to a study, for a focalized sylvan transmission of YFV to occur in a community of 130 monkeys, the minimal annual birth rate of 400 per 1000 would be necessary [228]. Whether or not this is sufficient for YFV persistence for a long period of quiescence in sylvan environments is unknown, because fertility of many primate species is about one per female per year and mortalities in newborns are not small. On the other hand, in the gallery forest in Africa, YF outbreak was recorded at intervals of only 3 years, despite the presence of only 10 monkeys. Thus, this is another source of enigma. Despite the importance, the necessary sizes of NHP populations for sylvan transmission of YFV have never been determined accurately. This also raises a question regarding heavy dependence only on non-immune primary hosts for the survival of YFV.

As for vectors, if they exclusively depend on primary NHPs for the source of bloodmeal, their survival becomes perilous when mortalities of infected NHPs are high, as in the Americas. In an experiment conducted in an atoll in the Pacific, when all vertebrates were eradicated, the established population of *Ae. albopictus* became extinct [229]. After all, female vectors can ill afford to discriminate immune from non-immune hosts for survival of the offsprings. In fact, it has been known that many hematophagous mosquitoes are opportunistic feeders and feed on non-essential hosts (incidental hosts) when primary hosts are scarce [217]. Thus, feeding on such incidental hosts as birds, bovine, rodents, opposums and horses by *Haemagogus* mosquitoes in South America may be interpreted to be a strategy of mosquitoes to satisfy the need of bloodmeal, given high mortalities of neotropical NHPs. Similarly in Africa, *Ae. furcifer* and *Ae. taylori* feed not only NHPs but on birds, rodents, and bats [230]. Interestingly, in the interface in Africa, two forms of a primary vector (*Ae. simpsoni*) evolved, one primatophilic and the other non-primatophilic.

For many investigators, both immune (primary) hosts and incidental hosts are thought to contribute to reduction in prevalence of disease incidence, a phenomenon known by “dilution effect.” A study on avian host diversity of West Nile virus, for example, confirmed this effect [231]. Thus, a concept of zooprophylaxis was conceived by some to be a possible means for a disease control. However, counterbalanced to this is a concept of host species diversity which ensures the survival of a disease agent [232].

In southeastern parts of Brazil, because the diversity of NHP fauna in fragmented forests is limited due to small space, it is thought that dependence of YFV transmission on a smaller number of NHP species (in particular howler monkeys) is intensified. Because mortalities of these monkeys are high, the importance of secondary NHPs (such as *Sapajus* monkeys), which are readily infected and propagate virus but whose mortalities are much lower, as bloodmeal sources increases. Even vaccinated humans are thought to contribute as sources of bloodmeal [233].

Nearly 90 years ago, Nelson C. Davis of the RF discovered that immune hosts still serve as a source of nourishment for the eggs of infective females and that these females still transmit virus in the next opportunities of blood feeding, because of multiple feeding behavior [234]. Furthermore, if females vertically transmit virus, feeding on immune hosts indirectly contribute to further transmission or maintenance of virus, however small this role may appear at first. Thus, although increase in the proportion of immune hosts definitely diminishes biological transmission according to the herd immunity concept [235], immune hosts still play a limited role in viral persistence at least indirectly. Thus, the recent conclusion [233] above is compatible with Nelson’s thought. Clearly, more research is necessary. Depending on the outcome, its impact on vaccination strategy based on herd immunity would not be insignificant.

## 9. Conclusions

As shown in the historical accounts above, many precautions by Manson and some of the preventive measures adopted in the international sanitary conventions must have played a significant role in preventing YF dispersal to Asia. Their merits have been rediscovered again in the wake of the COVID-19 pandemic in 2020, as exemplified by the revival of quarantine and disinfection practices, curtailing of docking of cruise ships arriving from coronavirus-infested countries, and source elimination. As predicted by Manson, even proliferation of unfounded rumors has been observed. Thus, including the importance in phylogeographic and epidemiologic studies, we can still learn valuable lessons from a variety of historical documents.

Nearly 40 years ago, Wilbur G. Downs pointed out multiple unknowns, deficiencies and other problematic issues on the ecology of YF [236]. Thereafter, thanks to the extensive investigations in the field and spectacular development of molecular virology by dedicated researchers around the world, many of the unknowns have been resolved; but YF absence in Asia, the mechanisms involved in viral spread and the dynamics of epidemics or epizootics, validity of cross-protection theory, and the factors regulating viral persistence in a given location have remained some of the important unknowns. Many of those unknowns require more comprehensive understanding of the importance of ecological factors. Those ecologic unknowns are another form of YF enigmas.

Fortunately, the importance of ecological factors is far more clearly recognized now in viral evolution, vector evolution, viral disease evolution, and in the evolution of cycling pattern of transmission [108,237,238,239,240]. For solving a variety of ecologic enigmas surrounding YFV, a holistic, integrated approach is necessary [4], as these subjects all represent an inter-related network of abiotic and biotic factors including virus-vector-host interaction and human activities. Recently, the next possible move of YFV to follow the paths of CHIKV and ZIKV out of Africa to Asia became a popular topic of discussion [241]. Elsewhere, the possibility of return of YF to North America, Europe, and the Caribbean areas has become a public health concern. Clearly, time is ripe to re-examine in depth this notion or expectation that because of sharing of a set of conditions, such as abundant infestation by competent *Ae. aegypti*, human population without immunity, and increased international human travel, YFV would similarly disperse and establish transmission in other receptive regions in the world.

## Figures and Tables

**Table 1:** Prevention of international yellow fever dispersal (1902–1944) with an emphasis on Asian concern.

Year	Event	Topics of Interest or Significance
1902	Establishment of the International Sanitary Bureau (later Pan American Health Organization), Washington, D.C.	This was the first international organization established for epidemic intelligence sharing, collaboration and assistance in preventing dispersal of major disease agents including yellow fever among American states.
1903	International Sanitary Conference, Paris	At this conference, the transmission mechanism of yellow fever based on the reports of the Walter Reed Commission was accepted, and YF was provisionally adopted as one of five diseases requiring prevention of dispersal. The conclusions of the conference emphasized establishing maritime regulation, sharing disease intelligence, and quarantine.
1905	Second International (Pan American) Sanitary Convention, Washington, D.C.	For the first time in history, international sanitary procedures and responsibilities of participating countries were codified. Prevention of YF spread was the principal objective. This code known by Pan American Sanitary Code was further revised until 1924 and served as model in all subsequent international conferences and in the International Health Regulation of the WHO.
1912	International Sanitary Conference, Paris	This conference updated the 1903 regulations, despite a marked decline of interest in quarantine and other measures. YF was officially adopted as a quarantinable disease.
1926	International Sanitary Conference, Paris	This conference established more refined maritime regulations. However, some countries retained the right to renounce the treatise after ratification, making the agreement meaningless.
1930	Eastern Bureau of the League of Nations meeting, Singapore	This conference conditionally prohibited direct airplane communication between Eastern (Asian) countries and yellow fever-endemic areas.
1932	International Convention for Aerial Navigation, Cape Town	In this convention, it was proposed to establish yellow fever “buffers” by means of “anti-amaryl aerodromes” in Africa and Asia (“amaril” meaning YF in French).
1933	International Sanitary Convention for Aerial Navigation, The Hague	This convention ratified the Aerial Navigation negotiated in Cape Town.
1944	International Sanitary Conference, Washington, D.C.	The 1926 maritime regulations were amended, and the 1933 aerial navigation was modified by dropping the requirement for “anti-amaryl aerodromes.” The requirement for yellow fever vaccination certification for international travel was adopted, and the conference more clearly defined “yellow fever-endemic areas.” Still, disinsection of aircraft for international flight was emphasized. Also, it designated seven yellow fever laboratories in Bogotá, Colombia; Rio de Janeiro, Brazil; International Health Division, Rockefeller Foundation, New York; Rocky Mountain Laboratory, Hamilton, Montana; Institut Pasteur, Paris; Entebbe, Uganda.
